# Permissible domain walls in monoclinic ferroelectrics. Part II. The case of *M_C_* phases

**DOI:** 10.1107/S2053273324002419

**Published:** 2024-04-29

**Authors:** Ido Biran, Semën Gorfman

**Affiliations:** a Department of Materials Science and Engineering, Tel Aviv University, Wolfson Building for Mechanical Engineering, Tel Aviv, 6997801, Israel; Czech Academy of Sciences, Czechia

**Keywords:** ferroelastic domains, monoclinic symmetry, X-ray diffraction

## Abstract

Monoclinic ferroelectric phases are prevalent in various functional materials, most notably mixed-ion perovskite oxides. These phases can manifest as regularly ordered long-range crystallographic structures or as macroscopic averages of the self-assembled tetragonal/rhombohedral nanodomains. The structural and physical properties of monoclinic ferroelectric phases play a pivotal role when exploring the interplay between ferroelectricity, ferroelasticity, giant piezoelectricity and multiferroicity in crystals, ceramics and epitaxial thin films. However, the complex nature of this subject presents challenges, particularly in deciphering the microstructures of monoclinic domains. In Paper I [Biran & Gorfman (2024). *Acta Cryst.* A**80**, 112–128] the geometrical principles governing the connection of domain microstructures formed by pairing *M_AB_* type monoclinic domains were elucidated. Specifically, a catalog was established of ‘permissible domain walls’, where ‘permissible’, as originally introduced by Fousek & Janovec [*J. Appl. Phys.* (1969), **40**, 135–142], denotes a mismatch-free connection between two monoclinic domains along the corresponding domain wall. The present article continues the prior work by elaborating on the formalisms of permissible domain walls to describe domain microstructures formed by pairing the *M_C_* type monoclinic domains. Similarly to Paper I, 84 permissible domain walls are presented for *M_C_* type domains. Each permissible domain wall is characterized by Miller indices, the transformation matrix between the crystallographic basis vectors of the domains and, crucially, the expected separation of Bragg peaks diffracted from the matched pair of domains. All these parameters are provided in an analytical form for easy and intuitive interpretation of the results. Additionally, 2D illustrations are provided for selected instances of permissible domain walls. The findings can prove valuable for various domain-related calculations, investigations involving X-ray diffraction for domain analysis and the description of domain-related physical properties.

## Abstract 2

Following the previous work [Biran & Gorfman (2024). *Acta Cryst.* A**80**, 112–128], all the possibilities for permissible (mismatch-free) walls between monoclinic domains of pseudocubic ferroelectric perovskites of *M_C_* type are analyzed. The study yields analytical expressions for the orientation of such walls, the orientation relationship between the lattice vectors and for the separation between Bragg peaks diffracted from matched domains.

## 1. Introduction

The orientation and properties of permissible domain walls (PDWs) connecting domains of monoclinic (*M*_*A*_/*M*_*B*_) symmetry were thoroughly discussed in our previous paper, denoted as Paper I (Biran & Gorfman, 2024 ▸). In addition to motivation for the exploration of monoclinic ferroelectric phases, we systematically derived the catalog of 84 PDWs, which included their corresponding Miller indices, the orientation relationship between crystallographic basis vectors and the separation between Bragg peaks diffracted from domains, connected along specific PDWs. Notably, we employed reasonable approximations to obtain analytical expressions for these quantities. We identified 48 PDWs of W-type and 36 PDWs of S-type, signifying whether their Miller indices are independent or dependent on the free lattice parameters. Moreover, we derived the specific combination of pseudocubic lattice parameters governing the orientation of the S-type domain walls as well as demonstrated how the change of a lattice parameter causes rotation of the domain wall.

According to Fu & Cohen (2000 ▸), Vanderbilt & Cohen (2001 ▸), Noheda *et al.* (1999 ▸, 2000 ▸), monoclinic ferroelectric phases (MFEP) can be categorized into *M*_*A*_/*M*_*B*_ or *M*_*C*_ types. These phases are distinguished by the permissible crystallographic direction of spontaneous polarization, if present, and the set of independent pseudocubic lattice parameters. Both types of MFEP are prevalent in ferroelectric perovskites and are frequently employed to describe the fine details of their crystallographic structures (see *e.g.* Guo *et al.*, 2003 ▸; Phelan *et al.*, 2015 ▸; Wang *et al.*, 2016 ▸; Gu *et al.*, 2014 ▸; Zhang *et al.*, 2011 ▸). Additionally, such phases are regularly reported in epitaxial thin films of ferroelectrics (Luo *et al.*, 2017 ▸; Bin Anooz *et al.*, 2022 ▸; de Oliveira Guimarães *et al.*, 2022 ▸; Wang *et al.*, 2022 ▸; Gaal *et al.*, 2023 ▸).

Paper I focused on MFEP of *M*_*A*_/*M*_*B*_ type only. The current article extends the same formalism to encompass monoclinic phases of the *M*_*C*_ type. Because the framework of this paper aligns closely with that of Paper I, most of the mathematical derivations have been provided in the supporting information. For a comprehensive list of notations, please refer to the corresponding section of the paper Gorfman *et al.* (2022 ▸) and Appendix *A* of Paper I.

## 2. Monoclinic ferroelectric phases: important definitions

### 2.1. The definition of the *M_C_* monoclinic phase

**Figure 1:**
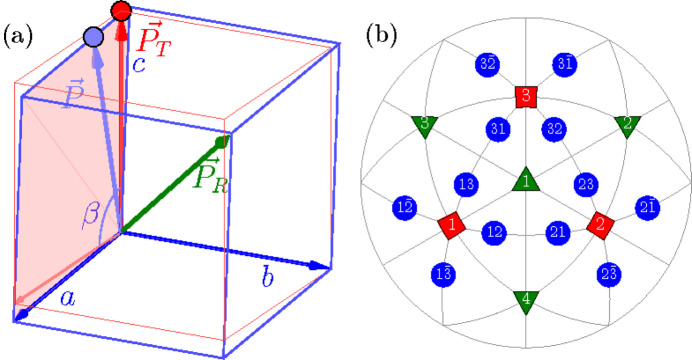
Schematic illustration of the *M*_*C*_ monoclinic domains. (*a*) The distortion of the unit cell and the rotation of the SPD. (*b*) The [111]-viewed stereographic projection, showing the SPDs for domains of tetragonal (red), rhombohedral (green) and monoclinic *M*_*C*_ (blue) symmetry. The tetragonal domains (1), (2), (3) correspond to the [100], [010] and [001] SPDs, respectively. Rhombohedral domains (1), (2), (3) and (4) correspond to the [111], 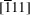 , 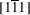 and 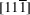 SPDs, respectively. These directions within the 12 monoclinic domains are further explained in Table 1 ▸.

The crystallographic structures of the *M*_*C*_ MFEP belong to the space-group types *Pm*, *Pc*. These structures are obtained through symmetry-lowering phase transitions from those described by the tetragonal (*T*) space-group types *P*4*mm*, *P*4*bm*. The ‘monoclinic’ mirror (*m*) /glide (*c*) plane aligns parallel to two edges of the pseudocubic unit cell. The space-group types *Pm*, *Pc* allow for the rotation of the spontaneous polarization direction (SPD) within this mirror plane. Additionally, these space groups permit any distortions of the unit cell that maintain the mirror plane. Fig. 1 ▸(*a*) provides a visual representation of both the distortion of the pseudocubic unit cell and possible rotation of the SPD.

#### 2.1.1. The directions of spontaneous polarization

The emergence of the *M*_*C*_ phase from the *T* phase prompts us to define the SPD through the slight rotation (by a small angle ρ) from one unit-cell edge such as [001] towards another such as [100]. This definition gives rise to distinct orientational variants of the monoclinic domains, denoted as *M*_*nm*_, where the first index *n* (*n* = 1–3) designates the SPD **T**_*n*_ in the ‘parent’ tetragonal domain with **T**_1_ = [100], **T**_2_ = [010], **T**_3_ = [001]. The second index *m* lists four options of monoclinic distortion |*m*| = 1–3 such that |*m*| ≠ *n*. For instance, the monoclinic domain *M*_12_ has its SPD rotated from [100] towards [010], whereas 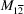 features rotation from [100] towards 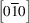 . Fig. 1 ▸(*b*) represents the stereographic projection, illustrating the potential SPDs in all 12 monoclinic domains.

In the following, we express the SPD relative to the axes of the Cartesian coordinate system, which are closely aligned with the pseudocubic basis vectors. For instance, in the case of the *M*_31_ domain we obtain

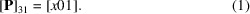

Herein, we introduce the notation

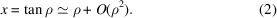




#### 2.1.2. Pseudocubic lattice parameters

Fig. 1 ▸(*a*) shows the *M*_*C*_ distortion of the pseudocubic unit cell. The corresponding pseudocubic lattice parameters *a*_*i*_, α_*i*_ (*i* = 1–3) are defined in terms of four independent variables: *a*, *b*, *c*, β. For instance, in the case of the *M*_31_ domain, we have 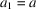 , 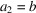 , 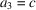 , 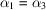 = 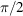 , 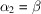 . The matrix of dot products corresponding to these lattice parameters is

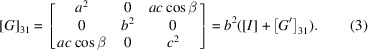

Here, [*I*] is the unitary matrix and

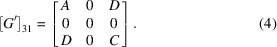

Assuming that the monoclinic distortions are small, *i.e.* keeping the first power of 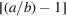 , 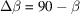 , 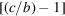 , we can write

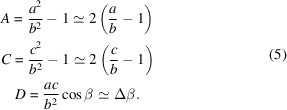

The resulting monoclinic crystal lattice is invariant with respect to *N*_*M*_ = 4 symmetry operations of the holohedry point group 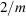 . The original cubic crystal lattice is invariant with respect to *N*_*C*_ = 48 operations of the holohedry point group *m*3*m*. Given that the monoclinic distortion can originate from any of these 48 equivalent variants, there are a total of 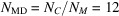 monoclinic domain variants. These domains are listed in Table 1 ▸, which includes domain identifiers, *M*_*nm*_, the [*G*′] metric tensors, the corresponding SPD as well as the lattice parameters *a*_1_, *a*_2_, *a*_3_, α_1_, α_2_, α_3_.

### 2.2. Domain pairs

**Figure 2:**
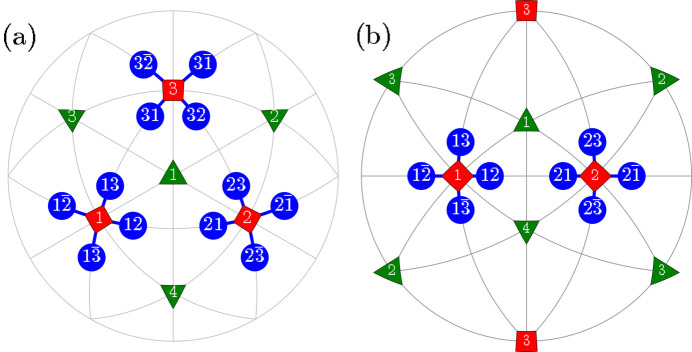
Schematic illustration of the ‘T-sibling-planar’ type of *M*_*C*_ monoclinic domain pairs. The term ‘T-sibling’ refers to the common *T* domain parent. The figure includes: (*a*) [111]-viewed stereographic projection, depicting SPDs within the 12 monoclinic domains. (*b*) [110]-viewed stereographic projection with the focus on the T-sibling pair types, originating from the tetragonal *T*_1_ and *T*_2_ domains.

Here we introduce different types of domain pairs, denoted as ‘T-sibling-planar’ (TSBP), ‘T-sibling-crossed’ (TSBC), ‘T-planar-1’ (TP1), ‘T-planar-2’ (TP2), ‘T-semi-planar’ (TSP), ‘T-semi-crossed’ (TSC) and ‘T-crossed’ (TC). The angles between the SPDs within each pair can be calculated using equation (2) and the third column of Table 1 ▸. A comprehensive summary of information pertaining to the domain pair types can be found in Table 2 ▸ (see Figs. 2 ▸ ▸ ▸ ▸ ▸ ▸–8 ▸).

## 3. The orientation of PDWs between different pairs of domains

The key steps for determining the orientation of the PDWs between two arbitrary domains (Table 1 ▸) have already been elucidated in the corresponding section of Paper I. These steps involve calculating the difference [*G*′]_*n*_ − [*G*′]_*m*_ and evaluating their respective eigenvalues and eigenvectors. As was done in Paper I, we present the detailed derivation for representatives of each domain pair type. However, for the sake of brevity, most technical details are provided in the supporting information.

### 3.1. PDWs connecting domain pairs of the type T-sibling-planar (TSBP)

Supporting information section S1.1 demonstrates the derivation of PDW orientation for the representative TSBP domain pair 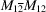 . It reveals that this pair of domains can be connected via two PDWs, each normal to the directions 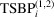 :

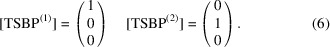




As in Paper I, the components of these vectors have the meaning of the Miller indices of the PDW plane. The Miller indices of both PDWs are independent of the lattice parameters. According to Fousek & Janovec (1969 ▸) these are *W-walls*.

### 3.2. PDWs connecting domain pairs of the type T-sibling-crossed (TSBC)

Supporting information section S1.2 demonstrates the derivation of PDW orientation for the representative TSBC domain pair *M*_12_ *M*_13_. It shows that this pair of domains may be connected along PDWs normal to the vectors 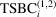 :

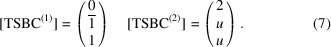




The wall, normal to [TSBC^(1)^], can be referred to as a W-wall. In contrast, the Miller indices of an *S-wall* [TSBC^(2)^] depend on the monoclinic distortion parameter *u*, as defined by

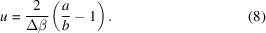

It is worth noting that, while both the numerator and denominator in equation (8) involve small monoclinic distortions, their ratio is generally not small. Specifically, *u* is strongly dependent on 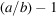 and Δβ. Remarkably, the orientation of this domain wall remains independent of the monoclinic lattice parameter *c*. Table 3 ▸ highlights cases in which the Miller indices of the S-wall are rational numbers.

### 3.3. PDWs connecting domain pairs of the type T-planar-1 (TP1)

Supporting information section S1.3 demonstrates the derivation of PDW orientation between the representative TP1 pair of domains *M*_12_ *M*_21_. It reveals the existence of two W-walls normal to the vectors 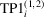 :

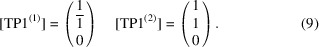




### 3.4. PDWs connecting domain pairs of the type T-planar-2 (TP2)

Supporting information section S1.4 demonstrates somewhat more intricate derivation of the PDW orientation between the representative TP2 types of domain pairs *M*_12_ and 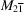 . The analysis reveals the existence of two PDWs, each normal to the vectors 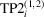 :

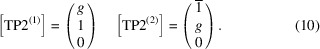




Here we introduced the notation

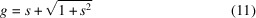

and

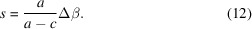

Both PDWs are S-walls. Their orientation appears to be significantly influenced by the lattice parameters *c*, *a* and Δβ but independent of *b*. Table 4 ▸ showcases two special/favorable cases in which these walls are perpendicular to the directions with rational Miller indices.

### 3.5. PDWs connecting domain pairs of the type T-semi-planar (TSP)

Supporting information section S1.5 presents the derivation of PDW orientation for the representative TSP type of domains *M*_31_ *M*_21_. As a result, two PDWs normal to the vectors 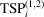 are identified:

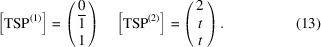




In this context, the following notation is employed:

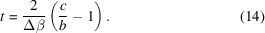

Table 5 ▸ lists special cases where the S-wall, normal to [TSP^(2)^], exhibits rational Miller indices. Notably, the wall’s orientation generally remains independent of the lattice parameter *a*.

### 3.6. PDWs connecting domain pairs of the type T-semi-crossed (TSC)

Supporting information section S1.6 provides the derivation of PDW orientation for the representative pair of TSP-type 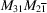 . The analysis reveals that this pair may connect via two PDWs, normal to the vectors 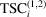 :

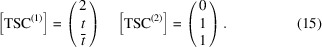




Similar to some cases discussed above, both W- and S-type domain walls (DWs) are present here. Favorable cases in which the [TSC^(1)^] S-wall exhibits rational Miller indices are contained in Table 5 ▸.

### 3.7. The absence of PDWs connecting domain pairs of the type T-crossed (TC)

Supporting information section S1.7 discusses the fact that generally no PDWs connecting domain pairs of the type TC exist.

## 4. The transformation matrices and the separation between Bragg peaks

Supporting information section S2 provides the derivation of the ‘Delta’ transformation matrices between the pseudocubic basis vectors of two domains *m* and *n*. These matrices [Δ*S*] are defined as follows:

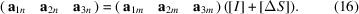




The methodology for deriving these matrices aligns with the procedure described in Paper I. These transformation matrices enable various domain-related calculations, such as precise calculation of the angles between the SPDs in the corresponding pair of domains connected along the relevant PDW. Most notably, the formalism provides expressions for the separation of Bragg peaks *HKL* diffracted from these domain pairs. This separation can be calculated using the matrix [ΔS*] between the corresponding reciprocal-lattice vectors:

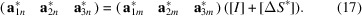




This leads to the expression for the splitting of the Bragg peaks, relative to the reciprocal coordinate system of domain *m*:

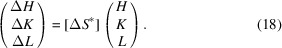

Such splitting is routinely measured in high-resolution single-crystal diffraction experiments (Gorfman & Thomas, 2010 ▸; Vergentev *et al.*, 2016 ▸; Zhang *et al.*, 2018 ▸; Choe *et al.*, 2018 ▸; Gorfman *et al.*, 2011 ▸, 2020 ▸, 2021 ▸, 2022 ▸). Therefore, expression (18) finds direct application in recognizing connected domain pairs within 3D diffraction patterns. Remarkably, when monoclinic distortion parameters (5) are small, both the elements of these transformation matrices as well as the components of the Bragg peak separation can be obtained analytically (see corresponding expressions in sections S2.1–S2.6).

## 5. Numerical examples

In this section we illustrate the principles underlying PDWs and the separation among the associated Bragg peaks, focusing on the domain pairs of TP1 and TP2 type. Given that these pairs have a common monoclinic twofold axis, we can illustrate the connection between such domains on the 2D drawings within the monoclinic mirror plane which is perpendicular to this axis [this plane is highlighted in Fig. 1 ▸(*a*)].

**Figure 9:**
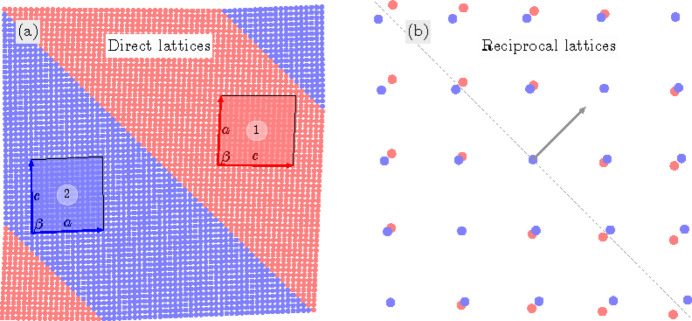
Illustration of a TP1-type pair of domains *M*_12_ (domain 1) and *M*_21_ (domain 2) connected along the (110) PDW. (*a*) Real-space illustration: domains are represented by the 2D lattices within the monoclinic mirror plane (this plane is highlighted in Fig. 1 ▸). The lattice nodes of domain 1 and domain 2 are marked by red and blue colors, respectively. The shaded parallelograms show enlarged sections of the pseudocubic unit cell with explicitly marked relevant lattice parameters. (*b*) Reciprocal-space illustration: *HK0* section of the reciprocal lattices of domains 1 and 2 (red and blue dots, respectively); the dashed line is parallel to the PDW, the arrow represents the PDW normal. The separation between the corresponding reciprocal-lattice points is along the PDW normal.

For the TP1 case we illustrate the connection between domains *M*_12_ *M*_21_. These domains have the lattice parameters *c* *a* *b* 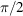 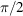 β and *a* *c* *b* 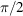 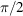 β, respectively (Table 1 ▸). For this numerical example, we assumed that 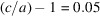 and β = 88°. According to (9) these domains connect along (110) or 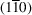 PDWs, both walls are normal to the mirror plane. Fig. 9 ▸(*a*) illustrates (110)-connection of these domains. Notably, these domains can self-organize into a lamella-type microstructure pattern, wherein *M*_12_ and *M*_21_ domains alternate periodically along the PDW normal. This arrangement introduces the concept of ‘adaptive’ phase as discussed by Jin *et al.* (2003 ▸), Viehland & Salje (2014 ▸). In this concept, the alternation and miniaturization of domains create states with macroscopic long-range periodicity and symmetry controlled by the volume ratios of the domains, rather than their lattice parameters only. Fig. 9 ▸(*a*) illustrates the possibility of such alternation while avoiding, however, the effects of domain miniaturization. Furthermore, Fig. 9 ▸(*b*) depicts the reciprocal lattices of these domains, clearly indicating that the separation between the Bragg peak diffracted from the corresponding matched domains occurs in the direction parallel to the PDW normal. It is worth noting that additional diffraction effects may emerge due to domain miniaturization and periodicity, as described by Wang (2006 ▸, 2007 ▸) in the case of tetragonal and rhombohedral nanodomains.

**Figure 10:**
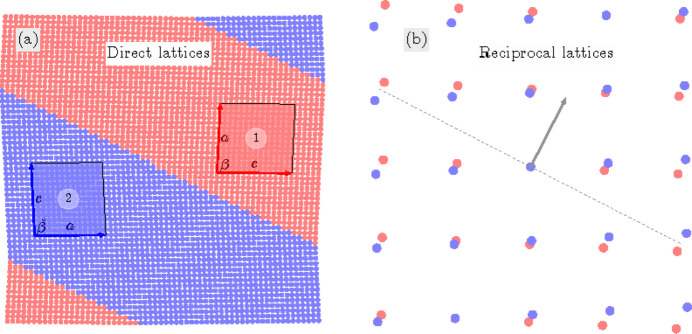
The same as Fig. 9 ▸ but for the case of a TP2 pair of domains *M*_12_ (domain 1) and 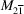 (domain 2) connected along the permissible (*g*10) domain wall. Remarkably, this PDW is an S-wall, *i.e.* the orientation of this wall depends on the free monoclinic lattice parameters, according to equation (10).

For the TP2 case we elucidate the connection between *M*_12_ and 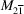 domains. These domains have the corresponding lattice parameters *c* *a* *b* 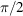 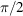 β and *a* *c* *b* 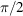 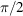 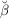 , respectively (Table 1 ▸). According to (10) these domains can form a connection along S-walls (*g*10) and 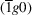 . In this specific numerical example, with 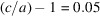 and β = 88°, we obtain that, according to (11) and (12), *g* ≃ 0.52. Fig. 10 ▸(*a*) illustrates the pairing of the 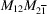 domains along the (*g*10) plane, like Fig. 9 ▸(*a*), while Fig. 10 ▸(*b*) provides visual representation of their corresponding reciprocal lattices. It is crucial to note that the orientation of this wall can vary with changes in lattice parameters.

## 6. Summarizing tables

The previous paragraphs and the supporting information outline the derivation of the equation for the PDWs’ Miller indices, orientation relationship between the lattice basis vectors, and the separation of Bragg peaks diffracted from the representative domain pairs. Similar equations can be derived for all the other pairs of domains. Tables and figures presented here list the corresponding quantities for all 84 existing PDWs. The full list includes:

12 PDWs connecting domain pairs of the type ‘T-sibling-planar’. All of them are W-walls.

24 PDWs connecting domain pairs of the type ‘T-sibling-crossed’. 12 of them are W-walls and another 12 of them are S-walls.

12 PDWs connecting domain pairs of the type ‘T-planar-1’. All of them are W-walls.

12 PDWs connecting domain pairs of the type ‘T-planar-2’. All of them are S-walls.

12 PDWs connecting domain pairs of the type ‘T-semi-planar’. Six of them are W-walls and another six of them are S-walls.

12 PDWs connecting domain pairs of the type ‘T-semi-crossed’. Six of them are W-walls and another six of them are S-walls.

Tables 6 ▸ ▸ ▸ ▸ ▸–11 ▸ contain the list of 84 PDW including 36 S- and 48 W-walls. Each table includes PDW number, the identifiers of the connected domains, the Miller indices of the corresponding PDW, the orientation relationship and the reciprocal-space separation between the Bragg peaks diffracted from this domain pair. In addition, the fifth column of these tables contains the approximate angle between the SPDs, providing zero or minimal domain wall charge, meaning that the SPDs (listed in Table 1 ▸) on both sides of the domain wall should have the same signs as the projection on the domain wall normal. For example, Table 6 ▸ shows that TSBP pair *M*_12_ and 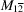 may connect along the PDWs parallel to either (100) or (010) lattice planes. According to Table 1 ▸ these domains contain polarization vectors parallel (or antiparallel) to the directions [1*x*0] or 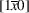 . The projection of these directions to the (100) normal is equal to 1 while the projection of these directions to the (010) plane normal is 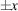 . Accordingly, the (100) DW should separate domains with the polarization vectors 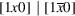 with the angle between them close to 0. In contrast, the (010) DW should separate domains with the polarization vectors 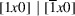 with the angle between them close to 180°.

Tables 6 ▸ ▸ ▸ ▸ ▸–11 ▸ reveal that certain W-walls have the same orientations. Table 12 ▸ presents all the distinct PDW orientations and their relevant details. It reveals that all the PDWs belong to five orientation families {100}, {110}, {2*uu*}, {*g*01}, {2*tt*}, so that PDWs of 45 distinct orientations are present. Furthermore, the table demonstrates the distribution of PDWs based on the pair type and the angle between the polarization directions.

**Figure 11:**
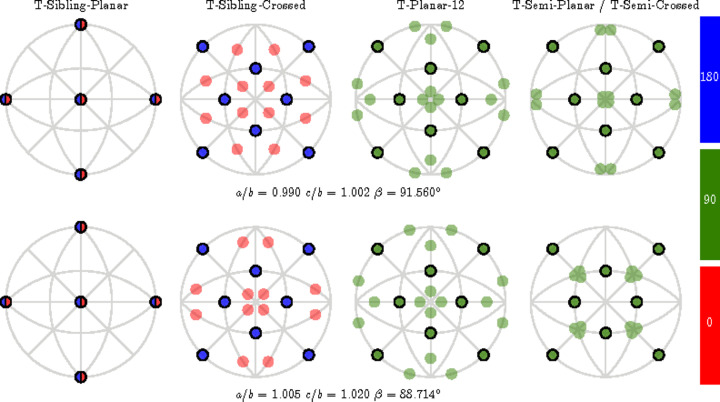
The directions of all PDW normals. There are a total of 45 distinct PDW orientations distributed among five orientation families. The normals are depicted using poles on the stereographic projection viewed along the [001] direction, with poles corresponding to the W-walls framed by solid lines. The lattice parameters are arbitrarily chosen.

Fig. 11 ▸ displays the orientation of all the PDWs for various choices of lattice parameters. The normals to these walls are depicted using the poles on the stereographic projection. W-walls are marked by poles with solid-line edges, and the color of the pole reflects the angle between SPDs, which is close to 0, 90 and 180° (as specified in Table 4 ▸).

## 7. Conclusion

In this study, we have applied the geometrical theory of permissible domains walls (PDWs) to compile a comprehensive list of 84 PDWs connecting ferroelastic domains of monoclinic *M*_*C*_ symmetry. Our list not only includes analytical expressions for the Miller indices of the PDWs but also matrices for transforming the corresponding pseudocubic basis vectors and formulas for calculating the reciprocal-space separation between corresponding Bragg peak pairs. These 84 PDWs encompass 45 different orientations and are organized into five distinct orientational families.

Our derivation of this extensive list is predicated on the assumption that the two-step transition from the cubic ( 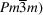 phase to the monoclinic (*Pm*/*Pc*) phase leads to the formation of 12 ferroelastic monoclinic domains. The first step of this transition, from the cubic 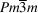 to the tetragonal *P*4*mm*/*P*4*bm* phase, results in the creation of three ferroelastic domains. In the second step, from the tetragonal *P*4*mm*/*P*4*bm* to the monoclinic *Pm*/*Pc* phase, each of these three domains divides into a group of four monoclinic domains. We have identified six distinct types of domain pairs, referred to as ‘T-sibling-planar’, ‘T-sibling-crossed’, ‘T-planar-1’, ‘T-planar-2’, ‘T-semi-planar’ and ‘T-semi-crossed’, each characterized by their own expression for the PDW orientation. As previously shown (Fousek & Janovec, 1969 ▸; Sapriel, 1975 ▸), we obtained that the Miller indices of PDWs can either remain fixed (W-walls) or depend on the values of the monoclinic lattice parameters (S-walls). Our investigation has revealed that the orientation of S-walls can be determined by three straightforward parameters, *u*, *g*, *t* with 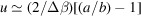 , 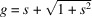 {here 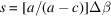 } and 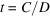 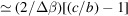 .

The results of our work (both the present one and the preceding one) can be useful in several ways. Firstly, the availability of simple analytical expressions for domain wall orientation aids in describing the domain switching through domain wall rotation or domain wall motion. Such a process can be initiated by a change in the temperature or the application of an external electric field, for example. Secondly, the formulas for calculating the separation between Bragg peaks (as found in Tables 6 ▸–11 ▸) can facilitate the study of monoclinic domain patterns, using single-crystal X-ray diffraction. Lastly, we provide expressions that may prove valuable for precisely calculating the angles between the spontaneous polarization directions of various domains.

## Supplementary Material

Supporting information. DOI: 10.1107/S2053273324002419/lu5034sup1.pdf

## Figures and Tables

**Figure 3:**
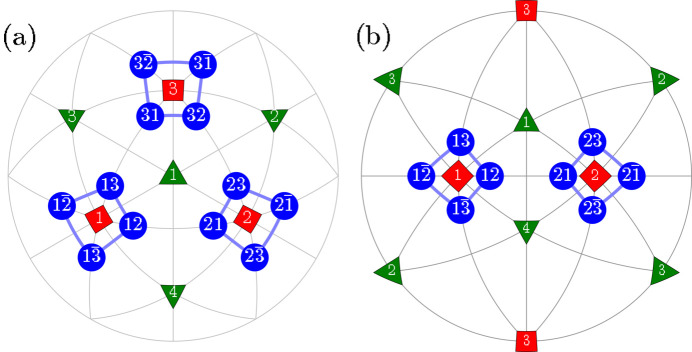
The same as Fig. 2 ▸ but for the case of *M*_*C*_ monoclinic domain pairs of the type ‘T-sibling-crossed’.

**Figure 4:**
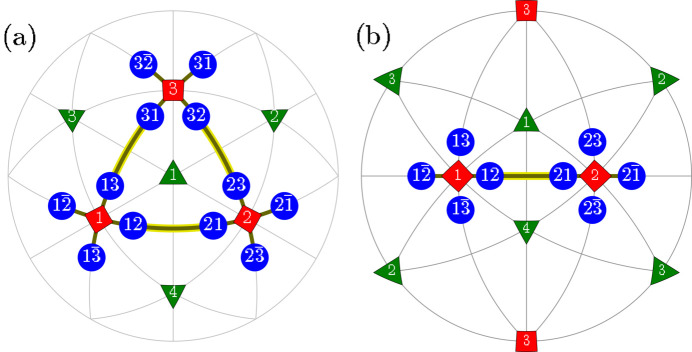
The same as Fig. 2 ▸ but for the case of TP1 (T-planar-1) type of *M*_*C*_ monoclinic domain pairs. The graphically overlapping connections (*e.g.* *M*_12_ *M*_21_ and 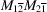 ) are drawn in different colors and line widths.

**Figure 5:**
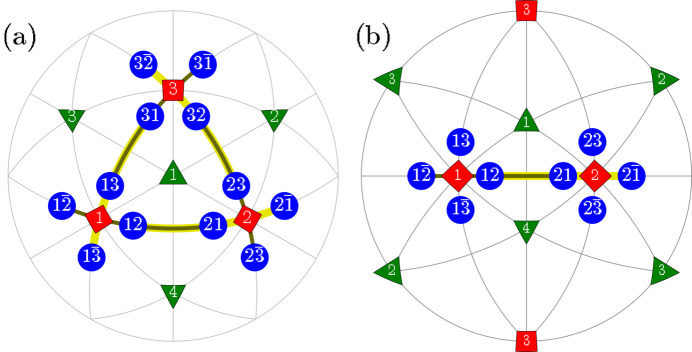
The same as Fig. 4 ▸ but for the case of domain pairs of the type TP2 (T-planar-2). As in Fig. 4 ▸, we used different colors and widths for graphically overlapping connections like 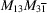 and 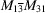 .

**Figure 6:**
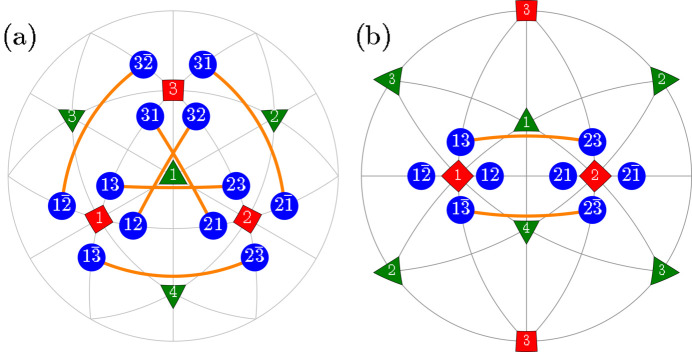
The same as Fig. 2 ▸ but for the case of *M*_*C*_ monoclinic domain pairs of the TSP (T-semi-planar) type.

**Figure 7:**
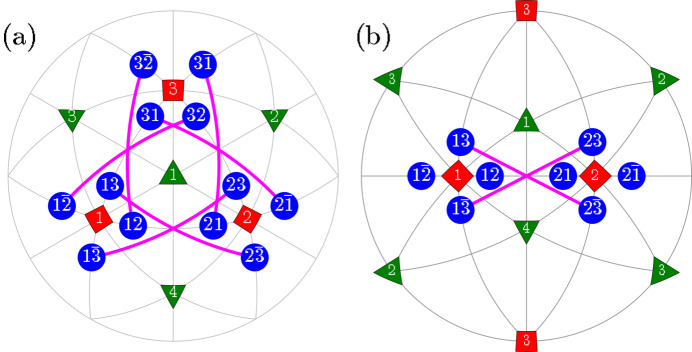
The same as Fig. 2 ▸ but for the case of *M*_*C*_ monoclinic domain pairs of the TSC (T-semi-crossed) type.

**Figure 8:**
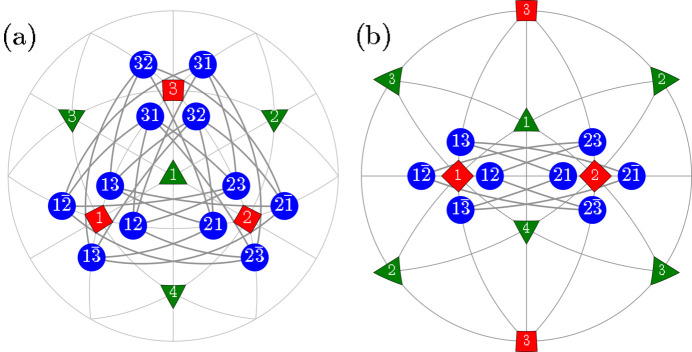
The same as Fig. 2 ▸ but for the case of *M*_*C*_ monoclinic domain pairs of the TC (T-crossed) type.

**Table 1:** Definition of the 12 monoclinic (*M*_*C*_ type) domains The first column contains the domain identifier as depicted in Fig. 1 ▸(*b*). The second column presents the twinning matrix [the definition of this matrix is explained by Gorfman *et al.* (2022 ▸) and also in the Appendix of Paper 1]. The third column provides the SPD for each domain, referenced to Cartesian axes of the parent cubic phase. The fourth column presents the pseudocubic lattice parameters expressed in terms of free parameters *a*, *b*, *c*, β. The notation 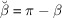 is used for brevity. The last column features the reduced matrix of dot products as defined by 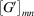 = 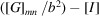 .

Domain name	Twinning matrix [*T*]	[**P**]_*mn*_	Pseudocubic Lp	[*G*′]_*mn*_
*M*_12_	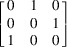	[1*x*0]	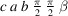	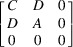
*M*_13_	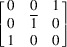	[10*x*]	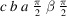	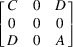
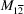	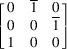	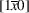	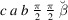	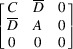
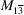	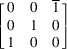	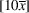	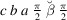	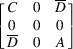
*M*_21_	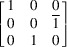	[*x*10]	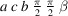	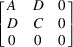
*M*_23_	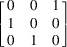	[01*x*]	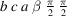	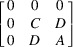
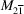	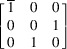	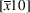	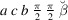	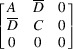
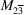	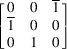	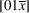	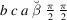	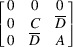
*M*_31_	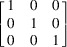	[*x*01]	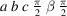	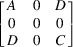
*M*_32_	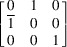	[0*x*1]	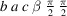	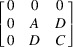
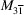	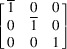	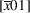	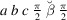	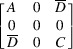
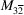	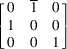	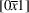	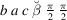	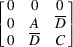

**Table 2:** Definitions of monoclinic *M*_*C*_ domain pair types The first and second columns provide the full and abbreviated names of the domain pairs. The third column presents the concise definition of each pair. The fourth column specifies the angle ξ between SPDs as a function of ρ (defined in Fig. 1 ▸). The fifth column indicates the number of corresponding domain pairs, while the last column references the corresponding figure.

Domain pair type full name	Short name	Formal definition	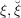	*N* pairs	Fig.
T-sibling-planar	TSBP	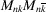	2ρ	6	Fig. 2 ▸
T-sibling-crossed	TSBC	*M*_*nk*_ *M*_*nl*_, |*k*| ≠ |*l*|	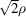	12	Fig. 3 ▸
T-planar-1	TP1	*M*_*nk*_ *M*_*ml*_ |*k*| = *m*, |*l*| = *n*, *kl* > 0	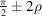	6	Fig. 4 ▸
T-planar-2	TP2	*M*_*nk*_ *M*_*ml*_ |*k*| = *m*, |*l*| = *n*, *kl* < 0	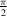	6	Fig. 5 ▸
T-semi-planar	TSP	*M*_*nk*_ *M*_*mk*_, *n* ≠ *m*	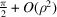	6	Fig. 6 ▸
T-semi-crossed	TSC	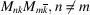	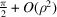	6	Fig. 7 ▸
T-crossed	TC	*M*_*nk*_ *M*_*ml*_ *n* ≠ *m*, |*k*| ≠ |*l*|,	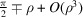	24	Fig. 8 ▸

**Table 3:** Special cases of *M*_*C*_ monoclinic distortion, leading to S-walls with rational Miller indices for TSBC domain pairs Column 1: relevant condition for the lattice parameters. Column 2: corresponding value of 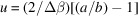 . Column 3: eigenvalue λ_3TSBC_ of the matrix [*G*′]_12_ − [*G*′]_13_. The case λ_3TSBC_ = 0 indicates complete lattice overlap between domains. Column 4: Miller indices of the DW.

Lattice parameters	*u*	λ_3TSBC_	S-wall orientation
*a* = *b*	0	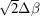	(100)
Δβ = 0	∞	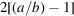	(011)
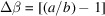	2	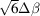	(111)

**Table 4:** Special cases of monoclinic distortion, leading to S-walls with rational Miller indices separating TP2 domain pairs The description of the columns is the same as for Table 3 ▸, just the third column contains the eigenvalue λ_3TP2_ of the matrix 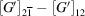 .

Lattice parameters	*s*	λ_3TP2_	[TP2^(1)^]-wall orientation	[TP2^(2)^]-wall orientation
Δβ = 0	0	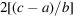	(110)	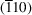
*c* = *a*	∞	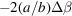	(100)	(010)

**Table 5:** The same as Table 3 ▸ just for the case of T-semi-planar types of domain pair Here the second column contains the special values of 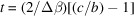 . The third column contains the eigenvalue of the matrix 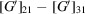 . The condition of mismatch-free connection is only relevant for the case if λ_3TSP_ ≠ 0 (otherwise the domain may connect along any plane).

Lattice parameters	*t*	λ_3TSP_	[TSP^(2)^]-wall orientation
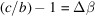	2	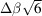	(111)
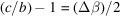	1	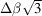	(211)
Δβ = 0	∞	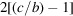	(011)
*b* = *c*	0	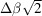	(100)
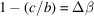	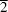	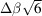	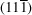
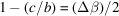	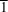	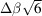	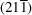

**Table 6:** Summary of 12 PDWs connecting domain pairs of the type T-sibling-planar Column 1: DW number. Columns 2 and 3: domain identifiers (per definitions in Fig. 1 ▸ and Table 1 ▸). Column 4: Miller indices of the DW. Column 5: angle between SPDs, corresponding to the condition of zero DW charge. Column 6: transformation matrix ([Δ*S*]) between the basis vectors of the domain *m*_1_ *n*_1_ to the basis vectors of the domain *m*_2_ *n*_2_. Column 7: separation between the Bragg peak with the indices *H*, *K*, *L* diffracted from these domains.

*N*	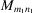	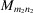	(*hkl*)	ξ (°)	[Δ*S*] (2Δβ)	[Δ**B**] (2Δβ)
1	*M*_12_	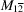	(100)	0	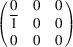	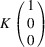
2	*M*_12_	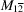	(010)	180	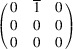	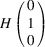
3	*M*_13_	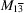	(001)	180	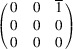	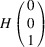
4	*M*_13_	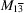	(100)	0	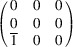	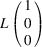
5	*M*_23_	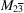	(010)	0	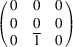	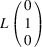
6	*M*_23_	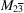	(001)	180	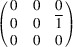	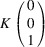
7	*M*_21_	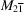	(100)	180	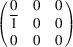	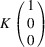
8	*M*_21_	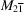	(010)	0	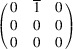	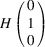
9	*M*_31_	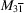	(001)	0	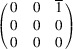	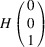
10	*M*_31_	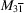	(100)	180	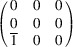	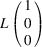
11	*M*_32_	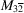	(010)	180	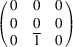	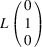
12	*M*_32_	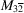	(001)	0	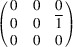	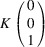

**Table 7:** The same as Table 6 ▸ but for PDWs connecting domain pairs of the type T-sibling-crossed

*N*	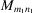	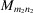	(*hkl*)	ξ (°)	[Δ*S*] ( 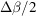 )	[Δ**B**] ( 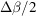 )
13	*M*_12_	*M*_13_	(2*uu*)	0	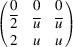	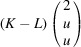
14	*M*_12_	*M*_13_	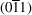	180	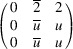	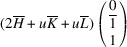
15	*M*_12_	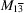	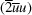	0	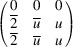	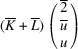
16	*M*_12_	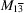	(011)	180	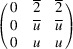	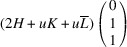
17	*M*_13_	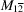	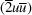	0	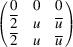	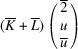
18	*M*_13_	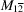	(011)	180	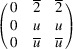	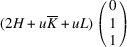
19	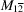	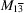	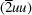	0	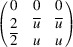	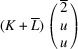
20	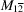	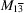	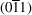	180	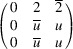	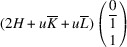
21	*M*_23_	*M*_21_	(*u*2*u*)	0	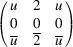	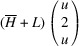
22	*M*_23_	*M*_21_	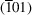	180	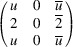	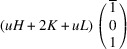
23	*M*_23_	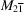	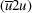	0	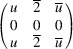	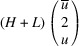
24	*M*_23_	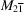	(101)	180	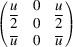	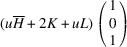
25	*M*_21_	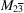	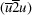	0	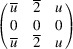	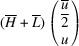
26	*M*_21_	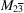	(101)	180	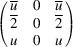	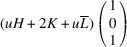
27	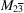	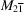	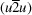	0	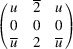	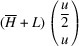
28	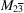	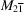	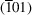	180	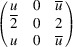	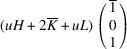
29	*M*_31_	*M*_32_	(*uu*2)	0	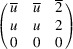	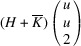
30	*M*_31_	*M*_32_	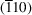	180	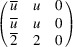	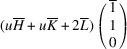
31	*M*_31_	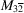	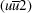	0	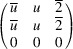	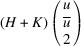
32	*M*_31_	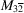	(110)	180	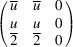	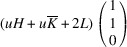
33	*M*_32_	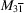	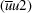	0	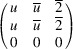	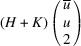
34	*M*_32_	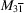	(110)	180	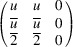	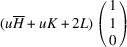
35	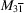	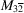	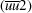	0	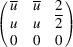	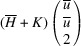
36	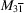	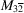	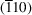	180	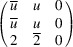	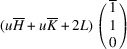

**Table 8:** The same as Table 6 ▸ but for PDWs connecting domain pairs of the type T-planar-1

*N*	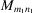	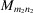	(*hkl*)	ξ (°)	[Δ*S*] 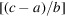	[Δ**B**] 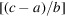
37	*M*_12_	*M*_21_	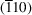	90	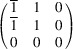	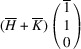
38	*M*_12_	*M*_21_	(110)	90	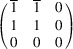	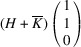
39	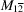	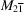	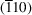	90	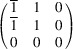	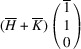
40	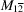	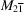	(110)	90	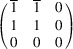	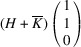
41	*M*_13_	*M*_31_	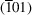	90	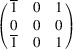	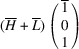
42	*M*_13_	*M*_31_	(101)	90	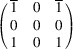	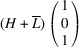
43	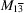	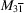	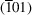	90	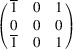	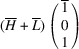
44	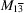	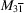	(101)	90	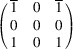	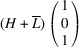
45	*M*_23_	*M*_32_	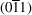	90	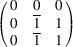	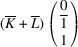
46	*M*_23_	*M*_32_	(011)	90	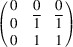	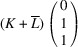
47	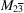	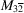	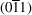	90	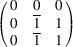	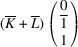
48	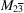	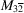	(011)	90	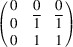	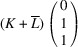

**Table 9:** The same as Table 6 ▸ but for PDWs connecting domain pairs of the type T-planar-2

*N*	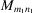	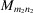	(*hkl*)	ξ (°)	[Δ*S*] 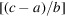	[Δ**B**] 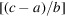
49	*M*_12_	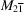	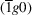	90	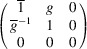	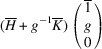
50	*M*_12_	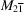	(*g*10)	90	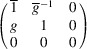	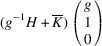
51	*M*_13_	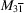	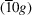	90	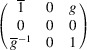	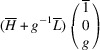
52	*M*_13_	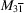	(*g*01)	90	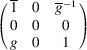	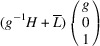
53	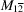	*M*_21_	(1*g*0)	90	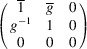	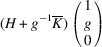
54	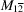	*M*_21_	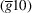	90	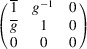	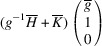
55	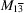	*M*_31_	(10*g*)	90	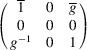	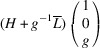
56	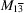	*M*_31_	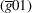	90	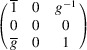	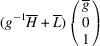
57	*M*_23_	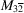	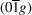	90	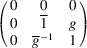	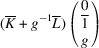
58	*M*_23_	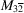	(0*g*1)	90	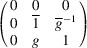	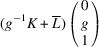
59	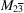	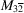	(01*g*)	90	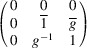	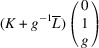
60	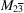	*M*_32_	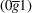	90	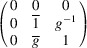	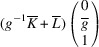

**Table 10:** The same as Table 6 ▸ but for PDWs connecting domain pairs of the type T-semi-planar

*N*	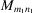	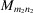	(*hkl*)	ξ (°)	[Δ*S*] 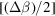	[Δ**B**] 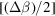
61	*M*_12_	*M*_32_	(*t*2*t*)	90	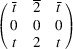	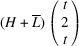
62	*M*_12_	*M*_32_	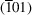	90	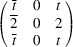	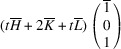
63	*M*_13_	*M*_23_	(*tt*2)	90	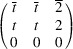	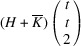
64	*M*_13_	*M*_23_	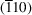	90	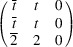	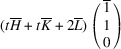
65	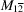	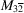	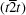	90	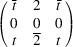	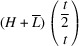
66	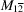	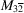	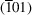	90	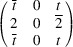	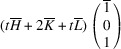
67	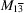	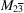	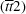	90	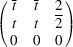	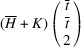
68	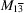	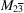	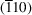	90	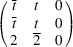	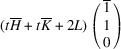
69	*M*_21_	*M*_31_	(2*tt*)	90	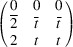	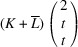
70	*M*_21_	*M*_31_	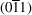	90	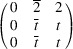	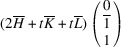
71	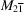	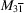	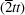	90	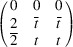	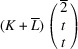
72	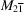	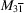	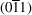	90	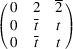	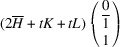

**Table 11:** The same as Table 6 ▸ but for PDWs connecting domain pairs of the type T-semi-crossed

*N*	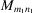	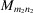	(*hkl*)	ξ (°)	[Δ*S*] 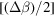	[Δ**B**] 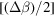
73	*M*_12_	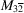	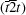	90	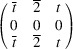	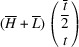
74	*M*_12_	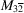	(101)	90	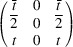	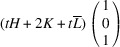
75	*M*_13_	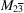	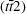	90	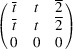	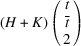
76	*M*_13_	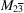	(110)	90	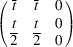	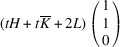
77	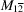	*M*_32_	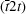	90	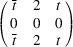	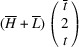
78	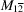	*M*_32_	(101)	90	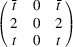	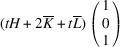
79	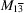	*M*_23_	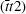	90	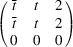	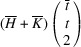
80	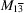	*M*_23_	(110)	90	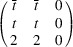	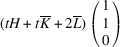
81	*M*_21_	*M*_31_	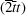	90	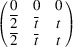	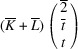
82	*M*_21_	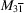	(011)	90	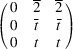	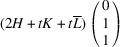
83	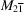	*M*_31_	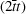	90	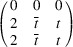	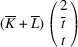
84	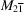	*M*_31_	(011)	90	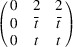	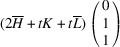

**Table 12:** Five orientation families of PDWs and their distribution between domain pairs of different types Column 1: identifier of the orientation family, with {} indicating the list of *m*3*m*-equivalent orientations. For example, {110} represents the list of (011), (101), (110), 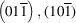 and 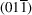 . Column 2: number of different orientations within the orientation family. Column 3: number of PDWs of the specific orientation family. The remaining columns: distribution of these PDWs according to pair type and the ‘zero-charge’ angle between polarization directions.

{*hkl*}	*M*	*N*	TSBP 0	TSBP 180	TSBC 0	TSBC 180	TP1 90	TP2 90	TSP 90	TSC 90
{100}	3	12	6	6	0	0	0	0	0	0
{110}	6	36	0	0	0	12	12	0	0	0
{2*uu*}	12	12	0	0	12	0	0	0	0	0
{*g*01}	12	12	0	0	0	0	0	12	0	0
{2*tt*}	12	12	0	0	0	0	0	0	6	6
All walls	45	84								
