# Supplementary material for: Permissible domain walls in monoclinic ferroelectrics. Part II. The case of MC phases
Source: Acta Crystallogr A Found Adv. 2024 Apr 29;80(Pt 3):293–304. doi: 10.1107/S2053273324002419 (PMC11067950; doi:10.1107/S2053273324002419)
Supplement: Supplementary file 1 [file a-80-00293-sup1.pdf]

FOUNDATIONS
ADVANCES

Volume 80 (2024)

Supporting information for article:

Permissible domain walls in monoclinic ferroelectrics. Part II: the case of MC phases

Ido Biran and Semën Gorfman

S1. The orientation of PDWs between various types of domains

This supporting material provides the derivation of the PDW orientation for the representative domain pair of each type. This derivation follows the algorithm introduced in (Gorfman *et al.*, 2022) and also implemented in Paper I. The key steps of this algorithm involve the calculation of eigenvalues $\lambda_1, \lambda_2, \lambda_3$ as well as eigenvectors of the matrix $[\Delta G'] = [G']_n - [G']_m$. The existence of the PDW, connecting the pair of domains is subject to one of these eigenvalues being zero, e.g., $\lambda_2 = 0$. In all the cases considered in this paper, this also means that $\lambda_1 = -\lambda_3$. The orthogonal matrix of eigenvectors $[V]$ enables the derivation of the normal to the PDWs using the expressions $V_{i1} \mp V_{i3}$ as well as being useful for the calculation of transformation matrices between the crystallographic basis vectors of the domains (these steps are further demonstrated in the supporting material S2).

S1.1. Permissible domain walls, connecting domain pairs of the type *"T-Sibling-Planar"* (TSBP).

Using the last column of Table 1 we obtain for the representative TSBP type pair $M_{1\bar{2}}\ M_{12}$:

$$[G']_{12} - [G']_{1\bar{2}} = \begin{pmatrix} 0 & 2D & 0 \\ 2D & 0 & 0 \\ 0 & 0 & 0 \end{pmatrix} = 2D[\Delta G'_{TSBP}] \tag{1}$$

with

$$[\Delta G'_{TSBP}] = \begin{pmatrix} 0 & 1 & 0 \\ 1 & 0 & 0 \\ 0 & 0 & 0 \end{pmatrix} \tag{2}$$

The eigenvalues of $[\Delta G'_{TSBP}]$ are $\bar{1}, 0, 1$. Accordingly, the eigenvalues of the matrix $[G']_{12} - [G']_{1\bar{2}}$ are $\bar{\lambda}_{3\ TSBP}, 0, \lambda_{3\ TSBP}$ so that:

$$\lambda_{3\ TSBP} = 2D \approx 2\Delta\beta \tag{3}$$

The orthogonal matrix of eigenvectors of $[\Delta G'_{TSBP}]$ (as well as of the matrix $[G']_{12} - [G']_{1\bar{2}}$) is

$$[V_{TSBP}] = \begin{pmatrix} -2^{-\frac{1}{2}} & 0 & 2^{-\frac{1}{2}} \\ +2^{-\frac{1}{2}} & 0 & 2^{-\frac{1}{2}} \\ 0 & 1 & 0 \end{pmatrix} \tag{4}$$

The PDWs normal to the vectors $TSBP_i^{(1)} \sim (V_{TSBP\,i1} - V_{TSBP\,i3})$and $TSBP_i^{(2)} \sim (V_{TSBP\,i1} + V_{TSBP\,i3})$ exist. Here

$$[TSBP^{(1)}] = \begin{pmatrix} 1 \\ 0 \\ 0 \end{pmatrix}$$
$$[TSBP^{(2)}] = \begin{pmatrix} 0 \\ 1 \\ 0 \end{pmatrix} \quad (5)$$

The Miller indices of both PDWs are independent of the lattice parameters. According to (Fousek & Janovec, 1969) these are *W-walls*.

S1.2. Permissible domain walls, connecting domain pairs of the type *"T-Sibling-Crossed"* (TSBC).

Using the last column of Table 1 we obtain for the representative TSBC type pair $M_{13}M_{12}$.

$$[G']_{12} - [G']_{13} = \begin{pmatrix} 0 & D & \bar{D} \\ D & A & 0 \\ \bar{D} & 0 & \bar{A} \end{pmatrix} = D[\Delta G'_{TSBC}] \quad (6)$$

with

$$[\Delta G'_{TSBC}] = \begin{pmatrix} 0 & 1 & \bar{1} \\ 1 & u & 0 \\ \bar{1} & 0 & \bar{u} \end{pmatrix} \quad (7)$$

and

$$u = \frac{A}{D} \approx \frac{2}{\Delta\beta}(\frac{a}{b} - 1) \quad (8)$$

The eigenvalues of the $[\Delta G'_{TSBC}]$ are $\bar{\lambda}_{TSBC}, 0, \lambda_{TSBC}$ with

$$\lambda_{TSBC} = \sqrt{2 + u^2} \quad (9)$$

The corresponding eigenvalues of the matrix $[G']_{12} - [G']_{13}$ are $\bar{\lambda}_{3\,TSBC}, 0, \lambda_{3\,TSBC}$ with

$$\lambda_{3\,TSBC} = D\sqrt{2 + u^2} \approx \Delta\beta\sqrt{2 + u^2} \quad (10)$$

The orthogonal matrix of eigenvectors of $[\Delta G'_{TSBC}]$ (as well as $[G']_{12} - [G']_{13}$) can be expressed as

$$[V_{TSBC}] = \frac{1}{2\lambda_{TSBC}} \begin{pmatrix} 2 & 2u & 2 \\ u - \lambda_{TSBC} & \bar{2} & u + \lambda_{TSBC} \\ u + \lambda_{TSBC} & \bar{2} & u - \lambda_{TSBC} \end{pmatrix} \tag{11}$$

Accordingly, the PDWs normal to the vectors $TSBC_i^{(1)} \sim (V_{TSBC\,i1} - V_{TSBC\,i3})$ and $TSBC_i^{(2)} \sim (V_{TSBC\,i1} + V_{TSBC\,i3})$ exist.

$$[TSBC^{(1)}] = \begin{pmatrix} 0 \\ \bar{1} \\ 1 \end{pmatrix}$$
$$[TSBC^{(2)}] = \begin{pmatrix} 2 \\ u \\ u \end{pmatrix} \tag{12}$$

The $[TSBC^{(1)}]$ can be referred to as *W-wall*. The Miller indices of $[TSBC^{(2)}]$ depend on the monoclinic distortion parameter u and therefore, according to (Fousek & Janovec, 1969) can be referred to as *S-wall*.

S1.3. Permissible domain walls connecting domain pairs of the type "***T-Planar-1***" ***(TP1)***.

Using the last column of Table 1 we obtain for the representative TP1-type pair $M_{12}\ M_{21}$.

$$[G']_{21} - [G']_{12} = \begin{pmatrix} A - C & 0 & 0 \\ 0 & C - A & 0 \\ 0 & 0 & 0 \end{pmatrix} = (C - A)[\Delta G_{TP1}] \tag{13}$$

with

$$[\Delta G_{TP1}] = \begin{pmatrix} \bar{1} & 0 & 0 \\ 0 & 1 & 0 \\ 0 & 0 & 0 \end{pmatrix} \tag{14}$$

The eigenvalues of $[\Delta G_{TP1}]$ are $\bar{1}, 0, 1$. The eigenvalues of $[G']_{21} - [G']_{12}$ are $\bar{\lambda}_{3\,TP1}, 0, \lambda_{3\,TP1}$ with

$$\lambda_{3\,TP1} = (C - A) \approx 2(\frac{c - a}{b}) \tag{15}$$

The orthogonal matrix of eigenvectors of both $[\Delta G_{TP1}]$ and $[G']_{21} - [G']_{12}$ is

$$[V_{TP1}] = \begin{pmatrix} 1 & 0 & 0 \\ 0 & 0 & 1 \\ 0 & 1 & 0 \end{pmatrix} \tag{16}$$

Accordingly, two W-walls normal to the vectors $TP1_i^{(1)}{\sim}(V_{TP1\,i1} - V_{TP1\,i3})$ and $TP1_i^{(2)}{\sim}(V_{TP1\,i1} + V_{TP1\,i3})$ exist.

$$[TP1^{(1)}] = \begin{pmatrix} 1 \\ \bar{1} \\ 0 \end{pmatrix}$$

$$[TP1^{(2)}] = \begin{pmatrix} 1 \\ 1 \\ 0 \end{pmatrix} \quad (17)$$

S1.4. Permissible domain walls connecting domain pairs of the type "*T-Planar-2*" (TP2).

Using the last column of Table 1 we obtain for the representative TP2 pair $M_{12}\ M_{2\bar{1}}$:

$$[G']_{2\bar{1}} - [G']_{12} = \begin{pmatrix} A-C & -2D & 0 \\ -2D & C-A & 0 \\ 0 & 0 & 0 \end{pmatrix} = (C-A)[\Delta G_{TP2}] \quad (18)$$

Here

$$[\Delta G_{TP2}] = \begin{pmatrix} \bar{1} & s & 0 \\ s & 1 & 0 \\ 0 & 0 & 0 \end{pmatrix}, \quad (19)$$

and

$$s = -\frac{2D}{(C-A)} \approx \frac{a}{(a-c)}\Delta\beta \quad (20)$$

The eigenvalues of $[\Delta G_{TP2}]$ can be found as $\bar{\lambda}_{TP2}, 0, \lambda_{TP2}$ with

$$\lambda_{TP2} = \sqrt{1+s^2} \quad (21)$$

Similarly, the eigenvalues of $[G']_{2\bar{1}} - [G']_{12}$ are $\bar{\lambda}_{3\,TP2}, 0, \lambda_{3\,TP2}$ with

$$\lambda_{3\,TP2} = 2(\frac{c-a}{b})\sqrt{1+s^2} \quad (22)$$

The orthogonal matrix of eigenvectors of $[\Delta G_{TP2}]$ (as well as $[G']_{2\bar{1}} - [G']_{12}$) is

$$[V_{TP2}] = \left(2\lambda_{TP2}(\lambda_{TP2}+1)\right)^{-\frac{1}{2}} \begin{pmatrix} -(\lambda_{TP2}+1) & 0 & s \\ s & 0 & (\lambda_{TP2}+1) \\ 0 & \left(2\lambda_{TP2}(\lambda_{TP2}+1)\right)^{\frac{1}{2}} & 0 \end{pmatrix} \quad (23)$$

Accordingly, two PDWs normal to the vectors $TP2_i^{(1)} \sim (V_{TP2\,i1} - V_{TP2\,i3})$ and $TP2_i^{(2)} \sim (V_{TP2\,i1} + V_{TP2\,i3})$ are possible.

$$[TP2^{(1)}] = \begin{pmatrix} g \\ 1 \\ 0 \end{pmatrix}$$
$$[TP2^{(2)}] = \begin{pmatrix} \bar{1} \\ g \\ 0 \end{pmatrix} \quad (24)$$

Here we introduced the notation:

$$g = s + \lambda_{TP2} \quad (25)$$

In derivation of (42) we considered that according to (39) $(1+\lambda_{TP2}-s)(\lambda_{TP2}+s) = (1+\lambda_{TP2}+s)$. It appears that both domain walls are *S-walls*, which means their orientation depends on the free monoclinic lattice parameters.

S1.5. Permissible domain walls connecting domain pairs of the type "*T-Semi-Planar*" (TSP).

Using the last column of Table 1 we obtain for the representative TSP-type domain pair $M_{31}M_{21}$ we obtain:

$$[G']_{21} - [G']_{31} = \begin{pmatrix} 0 & D & \bar{D} \\ D & C & 0 \\ \bar{D} & 0 & \bar{C} \end{pmatrix} = D[\Delta G_{TSP}] \quad (26)$$

Here, we introduced the following notation.

$$[\Delta G_{TSP}] = \begin{pmatrix} 0 & 1 & \bar{1} \\ 1 & t & 0 \\ \bar{1} & 0 & \bar{t} \end{pmatrix} \quad (27)$$

and

$$t = \frac{C}{D} \approx \frac{2}{\Delta\beta}\left(\frac{c}{b} - 1\right) \quad (28)$$

The eigenvalues and eigenvectors of $[\Delta G_{TSP}]$ can be written as $\bar{\lambda}_{TSP}, 0, \lambda_{TSP}$

$$\lambda_{TSP} = \sqrt{2 + t^2} \tag{29}$$

Accordingly, the corresponding eigenvalues of $[G']_{21} - [G']_{31}$ are $\bar{\lambda}_{3TSP}, 0, \lambda_{3TSP}$ with

$$\lambda_{3TSP} = \Delta\beta\sqrt{2 + t^2} \tag{30}$$

It is straightforward to see that the orthogonal matrix of eigenvectors of $[\Delta G_{TSP}]$ (as well as$[G']_{31} - [G']_{21}$) can be expressed as

$$[V_{TSP}] = \frac{1}{2\lambda_{TSP}}\begin{pmatrix} 2 & 2t & 2 \\ t - \lambda_{TSP} & \bar{2} & t + \lambda_{TSP} \\ t + \lambda_{TSP} & \bar{2} & t - \lambda_{TSP} \end{pmatrix} \tag{31}$$

Accordingly, two PDWs normal to the vectors $TSP_i^{(1)}$and $TSP_i^{(2)}$ exist.

$$[TSP^{(1)}] = \begin{pmatrix} 0 \\ \bar{1} \\ 1 \end{pmatrix}$$

$$[TSP^{(2)}] = \begin{pmatrix} 2 \\ t \\ t \end{pmatrix} \tag{32}$$

S1.6. Permissible domain walls connecting domain pairs of the type "*T-Semi-Crossed*" (TSC).

Using the last column of Table 1 we obtain for the representative pair $M_{3\bar{1}}\ M_{21}$ we obtain:

$$[G']_{21} - [G']_{3\bar{1}} = \begin{pmatrix} 0 & D & D \\ D & C & 0 \\ D & 0 & \bar{C} \end{pmatrix} = D[\Delta G_{TSC}] \tag{33}$$

Here we introduced the following notation.

$$[\Delta G_{TSC}] = \begin{pmatrix} 0 & 1 & 1 \\ 1 & t & 0 \\ 1 & 0 & \bar{t} \end{pmatrix} \tag{34}$$

The eigenvectors and eigenvalues of the $[\Delta G_{TSC}]$ can be found as $\bar{\lambda}_{TSC}$, 0, λ_{TSC}

$$\lambda_{TSC} = \sqrt{t^2 + 2} \tag{35}$$

Accordingly, the corresponding eigenvalues of $[G']_{21} - [G']_{3\bar{1}}$ are $\bar{\lambda}_{3\,TSC}, 0, \lambda_{3\,TSC}$ with

$$\lambda_{3\,TSC} = \Delta\beta\sqrt{t^2 + 2} \tag{36}$$

It is straightforward to see that the orthogonal matrix of eigenvectors of $[\Delta G_{TSC}]$ (as well as $[G']_{21} - [G']_{3\bar{1}}$) can be expressed as

$$[V_{TSC}] = \frac{1}{2\lambda_{TSC}}\begin{pmatrix} \bar{2} & 2t & 2 \\ \lambda_{TSC} - t & \bar{2} & \lambda_{TSC} + t \\ \lambda_{TSC} + t & 2 & \lambda_{TSC} - t \end{pmatrix} \tag{37}$$

Accordingly, two PDWs normal to the vectors $TSC_i^{(1)}$ and $TSC_i^{(2)}$ exist.

$$[TSC^{(1)}] = \begin{pmatrix} 2 \\ t \\ \bar{t} \end{pmatrix}$$

$$[TSC^{(2)}] = \begin{pmatrix} 0 \\ 1 \\ 1 \end{pmatrix} \tag{38}$$

As for the cases of DWs connecting domain pairs of the type "sibling" and "semi-planar", W-and S-type DWs are present here.

S1.7. Permissible domain walls connecting domains pairs of the type "*T-Crossed*" (TC).

We will show that the corresponding domain pairs of this type do not generally have any PDWs. Indeed, we can attempt to find such for the case of the representative pair of domains M_{13} M_{21}. According to the last column of Table 1 we get:

$$[G']_{13} - [G']_{21} = \begin{pmatrix} C - A & -D & D \\ -D & -C & 0 \\ D & 0 & A \end{pmatrix} \tag{39}$$

The determinant of $[G']_{13} - [G']_{21}$ can be calculated as

$$|\Delta G'_{\mathcal{B}}| = -(A - C)(D^2 - AC) \tag{40}$$

Accordingly, this pair of domains may connect along the PDW if one of the following conditions is fulfilled:

$$A = C \text{ or } a = c \tag{41}$$

or

$$D^2 = AC \tag{42}$$

These conditions are generally not fulfilled and therefore we can consider domain pairs of the type "crossed" are not compatible.

S2. Derivation of the transformation matrices and the separation between Bragg peaks

S2.1. General and simplified expressions

As described in (Gorfman *et al.*, 2022) and in the paper I, the transformation matrix $[\Delta S]$ between the basis vectors of two domains $(\boldsymbol{a}_{1n} \quad \boldsymbol{a}_{2n} \quad \boldsymbol{a}_{3n}) = (\boldsymbol{a}_{1m} \quad \boldsymbol{a}_{2m} \quad \boldsymbol{a}_{3m})([I] + [\Delta S])$ can be calculated using the following equation

$$[\Delta S] = [V][Z]\begin{pmatrix} 0 & 0 & y_1 \\ 0 & 0 & y_2 \\ 0 & 0 & 0 \end{pmatrix}[Z]^{-1}[V]^T \tag{43}$$

Here

$$[Z] = \begin{pmatrix} 1 & 0 & 0 \\ 0 & 1 & 0 \\ \pm 1 & 0 & 1 \end{pmatrix} \tag{44}$$

The sign $\pm$ before Z_{31} is used for the cases when PDW normal is $V_{i1} \mp V_{i3}$ respectively. The coefficients y_1 and y_2 can be calculated according to:

$$\begin{pmatrix} G_{m,11}^{(W)} & G_{m,12}^{(W)} \\ G_{m,21}^{(W)} & G_{m,22}^{(W)} \end{pmatrix}\begin{pmatrix} y_1 \\ y_2 \end{pmatrix} = \begin{pmatrix} G_{n,13}^{(W)} - G_{m,13}^{(W)} \\ G_{n,23}^{(W)} - G_{m,23}^{(W)} \end{pmatrix} \tag{45}$$

With $\left[G_{m,n}^{(W)}\right]$ being defined as

$$\left[G_{m,n}^{(W)}\right] = [Z]^T[Z] + [Z]^T[V]^T\left[G'_{m,n}\right][V][Z] \tag{46}$$

The equation (61) can be used to calculate the transformation matrix $[\Delta S]$ numerically or using a computer program. However, it also appears possible to obtain the elements of this matrix analytically. The simplified analytical expressions can be obtained assuming that the monoclinic distortion parameters introduced in the equations (*5*) are small and so, the second term of the equation (64) can be neglected compared to the first term. Then equation (61) can be re-written as follows

$$[\Delta S] = \pm\frac{\lambda_3}{2}[V]\begin{pmatrix} 1 \\ 0 \\ \pm 1 \end{pmatrix}(\mp 1 \quad 0 \quad 1)[V]^T \tag{47}$$

And the corresponding transformation matrix between the reciprocal lattice vectors
$(\boldsymbol{a}_{1n}^{*} \quad \boldsymbol{a}_{2n}^{*} \quad \boldsymbol{a}_{3n}^{*}) = (\boldsymbol{a}_{1m}^{*} \quad \boldsymbol{a}_{2m}^{*} \quad \boldsymbol{a}_{3m}^{*})([I] + [\Delta S^{*}])$

$$[\Delta S^{*}] = \pm\frac{\lambda_3}{2}[V]\begin{pmatrix}1\\0\\\mp 1\end{pmatrix}(\pm 1 \quad 0 \quad 1)[V]^{T} \tag{48}$$

The sign + or − before $\frac{\lambda_3}{2}$ is implemented for the cases of DW normal to $\boldsymbol{V}_{i1} - \boldsymbol{V}_{i3}$ and $\boldsymbol{V}_{i1} + \boldsymbol{V}_{i3}$ correspondingly. As is also easy to see that $[\boldsymbol{\Delta S}_{+}] = [\boldsymbol{\Delta S}_{-}]^{\boldsymbol{T}}$ and $[\boldsymbol{\Delta S}_{+}^{*}] = -[\boldsymbol{\Delta S}_{-}]$, $[\boldsymbol{\Delta S}_{-}^{*}] = -[\boldsymbol{\Delta S}_{+}]$. The transformation matrix $[\boldsymbol{\Delta S}^{*}]$ can be used to predict the splitting of Bragg peaks, diffracted from a pair of ferroelastic domains connected along the corresponding domain wall.

S2.2. The case of domain pairs of the type "*T-Sibling-Planar*"

We will apply (65) and (66) for the case PDW of the type *T-Sibling-Planar*. The corresponding transformation matrices $[\Delta S_{+}]$, $[\Delta S_{-}]$ are marked explicitly as $\left[\Delta S_{(100)}^{TSBP}\right]$ and $\left[\Delta S_{(010)}^{TSBP}\right]$. Using (21) we get that $\lambda_3 = 2\Delta\beta$. Substituting (22) into (65) we get:

$$\left[\Delta S_{(100)}^{TSBP}\right] = \left[\Delta S_{(010)}^{TSBP}\right]^{T} = 2\Delta\beta\begin{pmatrix}0 & 0 & 0\\1 & 0 & 0\\0 & 0 & 0\end{pmatrix} \tag{49}$$

Using (66) we can obtain the separation between the Bragg peak $H\ K\ L$ diffracted from the corresponding pair of domains as:

$$\left[\Delta B_{(100)}^{TSBP}\right] = -2\Delta\beta K\begin{pmatrix}1\\0\\0\end{pmatrix} \tag{50}$$

and

$$\left[B_{(010)}^{TSBP}\right] = -2\Delta\beta H\begin{pmatrix}0\\1\\0\end{pmatrix} \tag{51}$$

As mentioned in (Gorfman *et al.*, 2022) the separation between the Bragg peaks diffracted from pair of connected domains is parallel to the DW normal.

S2.3. The case of domain pairs of the type "*T-Sibling-Crossed*"

As in the case above the corresponding transformation matrices $[\Delta S_{+}]$, $[\Delta S_{-}]$ are then marked explicitly as $\left[\Delta S_{(0\bar{1}1)}^{TSBC}\right]$ and $\left[\Delta S_{(2uu)}^{TSBC}\right]$. According to (28), $\lambda_3 = \Delta\beta\sqrt{2+u^2}$. Substituting (29) into (65) we obtain:

$$\left[\Delta S^{TSBC}_{(0\bar{1}1)}\right] = \left[\Delta S^{TSBC}_{(2uu)}\right]^T = \frac{\Delta\beta}{2}\begin{pmatrix} 0 & 2 & \bar{2} \\ 0 & u & \bar{u} \\ 0 & u & \bar{u} \end{pmatrix} \tag{52}$$

Using (66) we get the following expression for the separation of Bragg peaks:

$$\left[\Delta B^{TSBC}_{(0\bar{1}1)}\right] = \frac{\Delta\beta}{2}(2H + uK + uL)\begin{pmatrix} 0 \\ \bar{1} \\ 1 \end{pmatrix} \tag{53}$$

and

$$\left[\Delta B^{TSBC}_{(2uu)}\right] = \frac{\Delta\beta}{2}(L - K)\begin{pmatrix} 2 \\ u \\ u \end{pmatrix} \tag{54}$$

S2.4. The case of domain pairs of the type "*T-Planar-1*"

We will mark the corresponding transformation matrices $[\Delta S_+], [\Delta S_-]$ explicitly as $\left[\Delta S^{TP1}_{(1\bar{1}0)}\right]$ and $\left[\Delta S^{TP1}_{(110)}\right]$. According to equation (33) we use $\lambda_3 = 2(\frac{c-a}{b})$. Substituting (34) into (65) we obtain:

$$\left[\Delta S^{TP1}_{(1\bar{1}0)}\right] = \left[\Delta S^{TP1}_{(110)}\right]^T = \frac{c-a}{b}\begin{pmatrix} \bar{1} & 1 & 0 \\ \bar{1} & 1 & 0 \\ 0 & 0 & 0 \end{pmatrix} \tag{55}$$

Using (66) we obtain the following expression for the separation of Bragg peaks:

$$\left[\Delta B^{TP1}_{(1\bar{1}0)}\right] = \frac{c-a}{b}(H + K)\begin{pmatrix} 1 \\ \bar{1} \\ 0 \end{pmatrix} \tag{56}$$

and

$$\left[\Delta B^{TP2}_{(110)}\right] = \frac{c-a}{b}(H - K)\begin{pmatrix} 1 \\ 1 \\ 0 \end{pmatrix} \tag{57}$$

S2.5. The case of domain pairs of the type "*T-Planar-2*"

We will mark the corresponding transformation matrices $[\Delta S_+], [\Delta S_-]$ explicitly as $\left[\Delta S^{TP2}_{(g10)}\right]$ and $\left[\Delta S^{TP2}_{(1\bar{g}0)}\right]$. According to equation (40) we use $\lambda_3 = 2\left(\frac{c-a}{b}\right)\lambda_{TP2}$. Substituting (41) into (65) we obtain:

$$\left[\Delta S^{TP2}_{(g10)}\right] = \left[\Delta S^{TP2}_{(1\bar{g}0)}\right]^T = \frac{c-a}{b}\begin{pmatrix}\bar{1} & -g^{-1} & 0\\ g & 1 & 0\\ 0 & 0 & 0\end{pmatrix} \tag{58}$$

Accordingly, we get the following expressions for the separation of the Bragg peaks:

$$\left[\Delta B^{TP2}_{(g10)}\right] = \frac{c-a}{b}\left(\frac{H}{g} - K\right)\begin{pmatrix}g\\ 1\\ 0\end{pmatrix} \tag{59}$$

and

$$\left[\Delta B^{TP2}_{(1\bar{g}0)}\right] = \frac{c-a}{b}\left(H - \frac{K}{g}\right)\begin{pmatrix}1\\ \bar{g}\\ 0\end{pmatrix} \tag{60}$$

S2.6. The cases of domain pairs of the type "*T-Semi-Planar*"

We will mark the corresponding transformation matrices $[\Delta S_+], [\Delta S_-]$ explicitly as $\left[\Delta S^{TSP}_{(0\bar{1}1)}\right]$ and $\left[\Delta S^{TSP}_{(2tt)}\right]$. According to equation (48) we use $\lambda_3 = \Delta\beta\sqrt{2+t^2}$. Substituting (49) into (65) we obtain:

$$\left[\Delta S^{TSP}_{(0\bar{1}1)}\right] = \left[\Delta S^{TSP}_{(2tt)}\right]^T = \frac{\Delta\beta}{2}\begin{pmatrix}0 & 2 & \bar{2}\\ 0 & t & \bar{t}\\ 0 & t & \bar{t}\end{pmatrix} \tag{61}$$

Using (66) we obtain the following expression for the separation of Bragg peaks:

$$\left[\Delta B^{TSP}_{(0\bar{1}1)}\right] = \frac{\Delta\beta}{2}(2H + tK + tL)\begin{pmatrix}0\\ \bar{1}\\ 1\end{pmatrix} \tag{62}$$

and

$$\left[\Delta B^{TSP}_{(2tt)}\right] = \frac{\Delta\beta}{2}(L - K)\begin{pmatrix}2\\ t\\ t\end{pmatrix} \tag{63}$$

The transformation matrices for the case of domain pairs of the type "*T-Semi-Crossed*" are derived identically.
